# Relationships of maternal hemodynamics in the third trimester with fetal umbilical artery doppler indices, estimated fetal weight and birth weight in women with and without congenital heart disease

**DOI:** 10.1002/ijgo.16190

**Published:** 2025-01-31

**Authors:** Francois Dos Santos, Ellen Barr, Philip J. Steer, Mark R. Johnson

**Affiliations:** ^1^ Imperial College London, Chelsea And Westminster Hospital London UK; ^2^ O&G Department Fiona Stanley Hospital Perth Western Australia Australia

**Keywords:** congenital heart disease, high‐risk pregnancy, impedance cardiography, maternal hemodynamics, umbilical artery doppler

## Abstract

**Objective:**

To compare differences in maternal hemodynamics, measured non‐invasively by impedance cardiography and mean arterial blood pressure (MAP)—at rest and with high‐intensity exercise—between pregnant women with corrected congenital heart disease (CHD) and low‐risk (LR) pregnant controls, and to correlate these findings with umbilical artery Doppler in the third trimester, estimated fetal weight (EFW) and birth weight (BW).

**Methods:**

Prospective longitudinal study with hemodynamic exercise studies and fetal ultrasound between 30 and 34 weeks' gestation. Approval was obtained from London South East Research Ethics Committee.

**Results:**

There were no differences in heart rate (HR), stroke volume (SV), or cardiac output (CO) at rest between the two groups. HR at peak exercise was significantly lower in the CHD group, and MAP was significantly higher at rest and immediately after exercise. In the CHD group there was a significant association between CO at peak exercise and BW. In the LR group there was a significant association between peak CO with exercise and the abdominal circumference/EFW ratio and between HR at rest and BW. There were no differences in the Doppler indices between groups. There was a statistically significant association between uterine artery Doppler pulsatility index and BW in the LR group, but not in the CHD group.

**Conclusions:**

There was no difference between Doppler indices in the third trimester between a LR population and a population with corrected CHD with no or minimal functional impairment. This suggests that factors other than defective placentation may be causing the lower BW in the CHD population.

## Synopsis

Pregnant women with CHD have an increased risk of obstetric complications such as FGR. No differences were found between Doppler indices between low‐risk women and women with CHD.

## 1 INTRODUCTION

Pregnancy in women with congenital heart disease (CHD) is associated with increased maternal cardiovascular and obstetric complications with fetal morbidity, such as small for gestational age (SGA) and fetal growth restriction (FGR).^1^, ^2^, ^3^, ^4^ The cardiovascular complications are reported to be associated with blunted cardiovascular adaptation to pregnancy in women with CHD, although longitudinal data are scarce.^1^, ^5^

The high risk of adverse pregnancy and cardiac events in pregnant women with CHD is mainly due to the strain that pregnancy imposes on the cardiovascular system: an increase in blood volume, heart rate (HR), stroke volume (SV), and cardiac output (CO).^2^ CO is a key hemodynamic parameter in pregnancy as it is related to placental perfusion.

Placentation can directly or indirectly influence cardiovascular adaptation to pregnancy and it has been demonstrated that FGR is associated with lower CO and higher total vascular resistance (TVR).^6^, ^7^ In women with CHD, it is postulated that impaired hemodynamic adaptation can lead to inadequate placentation (rather than the reverse) and explain the higher incidence of FGR.^8^

Assessing hemodynamic parameters in the third trimester and their association with birth weight (BW) and BW centiles could provide an insight into how hemodynamic maladaptation influences growth.

The main objectives of this study were to compare differences in maternal hemodynamics—HR, SV, CO, and mean arterial blood pressure (MAP)—at rest and with high‐intensity exercise—between pregnant women with corrected CHD and low‐risk (LR) pregnant controls. We correlated these findings with Doppler flow velocity waveform indices of uterine artery Dopplers (UtAD), umbilical artery Dopplers (UAD) and middle cerebral artery (MCA) Dopplers obtained in the third trimester (between 30 and 32 weeks of gestation), estimated fetal weight (EFW) at that time, and BW.

## 2 METHODS

Participants were recruited from LR pregnancy booking clinics and from the Joint Obstetric Cardiac Medicine Clinic at the Chelsea and Westminster Hospital NHS Foundation Trust, London, UK. Inclusion criteria were: pregnant women with a singleton pregnancy ≥18 years of age and structural CHD (CHD group), and LR women with a singleton pregnancy, ≥18 years of age, no history or symptoms of heart disease, and no known chronic medical comorbidities (LR group). Between 30 and 34 weeks' gestation, a fetal ultrasound for EFW and fetal Doppler studies (UtAD, UAD and MCA Doppler) were performed, with measurement of maternal blood pressure (BP) and hemodynamics using impedance cardiography (ICG). In the CHD group, only those having an ultrasound as part of standard NHS care were included and, therefore, UtAD measurements were not performed in all participants. For the LR group, this was an extra visit (outside standard care), and measurements and Doppler studies were obtained in all cases. All participants provided written informed consent. This study was approved by the Health Research Authority and the London South East Research Ethics Committee (REC reference: 17/LO/0970).

### 2.1 Ultrasound measurements

The GE Voluson™ S10 Expert (General Electric Company, Boston, Massachusetts, USA) equipped with a transabdominal C4‐8‐D probe (frequency 8–2 MHz) was used to perform the scans. Measurements of the head circumference, abdominal circumference (AC), and femur length were obtained to calculate the EFW using the Hadlock formula according to International Society of Ultrasound in Obstetrics and Gynecology (ISUOG) guidelines.^9^ Following fetal biometry, the Doppler studies were performed according to ISUOG guidelines.^10^

### 2.2 Hemodynamic data

Participants were asked to rest for 5 minutes and a BP measurement was taken using an Omron® MIT Elite device (Omron Healthcare, Kyoto, Japan), an automated device using inflationary oscillometry, validated for use in pregnancy.^11^, ^12^ After this, six PF‐50™ AgCl electrodes were positioned according to the ICG device manufacturer's instructions (PhysioFlow® Enduro, Manatec Biomedical, Poissy, France), connected to the device, and continuous measurements of HR, SV, and CO were performed. Readings took place at rest (T1) and at peak exercise (T2)—participants were asked to exercise on an ergometer cycle (Kettler E3 Ergometer Cycle; Kettler GmbH, Ense‐Parsit, Germany) to 70% of predicted maximum capacity for age, calculated as (220 –age) × 0.7. The cycle had an operator‐regulated resistance of 25–4000 W and gravity pedals. Participants were informed of the target HR and were given the freedom (time and power usage) to achieve that HR. During the exercise, they were given feedback on their HR and instructed to increase the intensity of the exercise to achieve the target HR. Once the target HR was achieved, they were instructed to maintain it for 1 minute, immediately after which the T2 measurement took place. BP was measured immediately after the participants transferred from the exercise cycle ergometer to a left lateral position. A percentage increase in HR, SV, and CO was calculated as: ΔHR = ([HR_peak exercise_ − HR_rest_]/HR_peak exercise_) × 100%; ΔSV = ([SV_peak exercise_ − SV_rest_]/SV_peak exercise_) × 100% and ΔCO = ([CO_peak exercise_ − CO_rest_]/CO_peak exercise_) × 100%, as an indirect measurement of cardiac function. MAP was calculated as: diastolic BP + (systolic BP−diastolic BP)/3.^13^

### 2.3 Statistical analysis

Statistical analyses were performed using IBM SPSS Statistics, version 26.0, 2019 (IBM Corp., Armonk, NY, USA) and Microsoft Excel for Mac, version 16.30, 2019 (Microsoft Corp., Redmond, WA, USA). The Kolmogoroff–Smirnoff and Shapiro–Wilk tests were used to assess normality of the distribution of the data as well as visual inspection of the histograms and Q–Q′ normality plots. Maternal characteristics were compared using chi‐squared or Mann–Whitney *U*‐test, as appropriate. Spearman rank correlation (*r*_s_) was used to evaluate the correlation between EFW, AC/EFW, BW, and BW centile with hemodynamic parameters at rest and at peak exercise. Doppler indices were presented as median (min–max). Spearman‐rank correlation (*r*_s_) was also used to evaluate correlations between EFW, AC/EFW ratio, BW, and BW centile, hemodynamic parameters with all Doppler indices, and *P*‐values were adjusted for multiple comparisons using Bonferroni correction. Doppler indices were adjusted for gestational age using a linear mixed model.

### 2.4 Sample size

Sample size calculations were performed using the G*Power software (G*Power for Mac Os X, v. 3.1, February 2020; Heinrich‐Heine‐Universität, Dusseldorf, Germany). For the association between Doppler indices and BW, to achieve a similar result to that of Bamfo et al.^14^ with an alpha  = 0.05 and a 95% power, 58 LR subjects and 17 subjects in the CHD group were required.

## 3 RESULTS

### 3.1 Maternal characteristics and obstetric and fetal outcomes

Table 1 describes the characteristics and obstetric outcomes of the participants. We included 62 LR individuals and 16 women with CHD. BW centiles were calculated using the Aberdeen Maternity and Neonatal Databank nomograms.^15^ Specific cardiac lesions of the participants with CHD are included at the bottom of Table 2.

**TABLE 1:** Maternal characteristics and obstetric outcomes of low‐risk (LR) pregnant women and pregnant women with congenital heart disease (CHD) included in the study of maternal hemodynamics and association with birth weight and fetal Doppler indices. Significance level: *P* < 0.05.

	LR (*N* = 62)	CHD (*N* = 16)	*P*‐value
Age (y) (median [min–max])	33 (24–42)	30 (25–39)	0.009**
Race (*n* [%])			0.64
White	51 (82.3)	14 (87.5)	
Non‐white	11 (17.7) (2 black, 5 Asian, 2 Middle‐Eastern, 2 mixed)	2 (12.5) (1 Asian, 1 Middle‐Eastern)	
Weight (kg) (median [min–max])	62.0 (43.0–100.0)	69.0 (53.0–101.0)	0.010**
BMI (median [min–max])	22.5 (18.0–34.0)	26.5 (17.6–34.9)	0.038*
Smoking (*n* [%])			0.96
Never smoked	55 (88.7)	14 (87.5)	
Current/past smoker	7 (11.3)	2 (12.5)	
Parity (*n* [%])			0.98
P0	44 (71.0)	11 (68.8)	
*P* ≥ 1	18 (29.0)	5 (31.2)	
Mode of delivery (*n* [%])			0.23
Spontaneous vaginal delivery	30 (48.4)	6 (37.5)	
Ventouse vaginal delivery	2 (3.2)	0 (0.0)	
Forceps vaginal delivery	6 (9.7)	1 (6.3)	
Emergency cesarean section	16 (25.8)	3 (31.3)	
Elective cesarean section	8 (12.9)	4 (25.0)	
Mode of delivery (*n* [%])			0.21
Vaginal (all, including instrumental)	38 (61.3)	7 (43.8)	
Cesarean section (all)	24 (38.7)	9 (56.2)	
Gestational age at delivery (wk) (median [min–max])	39 + 6 (35 + 6–42 + 0)	38 + 5 (34 + 3–41 + 0)	0.001***
Sex of the baby (*n* [%])			0.89
Female	26 (41.9)	7 (43.8)	
Male	36 (58.1)	9 (56.2)	
Birth weight (g) (median [min–max])	3435 (2360–5220)	2915 (1970–3855)	0.009**
Birth weight centile (%) (median [min–max])	60.0 (3.0–100.0)	31.0 (7.0–92.0)	0.050*
Estimated blood loss (mL) (median [min–max])	400 (100–2005)	480 (300–800)	0.52

*Note*: *P < 0.05; **P < 0.01; ***P < 0.001.

**TABLE 2:** Specific cardiac lesions of participants with congenital heart disease included in the study (*N* = 16).

CHD lesion	*N*	Use of medication
rTOF	5	One participant on LDA, two participants on beta‐blockers
rTOF only	4	
rTOF + rSA stenosis	1	
rCoA	2	
rCoA only	1	
rCoA + BAV	1	
rTGA	3	One participant on beta‐blockers, one participant on LMWH
rTGA (arterial switch)	2	
rTGA (atrial switch)	1	
Fontan TCPC	1	On LMWH
Ebstein's anomaly	1	
Right hemi‐truncus	1	
Mixed lesions	3	
AS + BAV	1	
rVSD + rPDA + BAV	1	
BAV	1	

Abbreviations: AS, aortic stenosis; BAV, bicuspid aortic valve; CoA, coarctation of the aorta; LDA, low‐dose aspirin; LMWH, low‐molecular‐weight heparin; PDA, patent ductus arteriosus; r, repaired; SA, subaortic stenosis; ToF, tetralogy of Fallot; TCPC, total cavopulmonary connection; TGA, transposition of great arteries; VSD, ventricular septal defect.

There were no differences in HR, SV, or CO at rest between the two groups. HR at peak exercise was significantly lower (*P* = 0.006) in the CHD group but there were no differences in SV or CO at peak exercise. MAP was significantly higher at rest and immediately after exercise in the CHD cohort (Table 3).

**TABLE 3:** Hemodynamic parameters in the two studied populations.

	LR (*N* = 63)		CHD (*N* = 16)		*P*	
HR rest (bpm)	83 (60–106)		83 (68–99)		0.75	
SV rest (mL)	75.9 (45.2–102.2)		85.7 (55.4–101.1)		0.25	
CO rest (L/min)	6.1 (4.3–9.9)		6.4 (4.4–8.1)		0.27	
MAP rest (mmHg)	67.5 (52.3–92.3)		69.3 (57.3–85.3)		0.045*	
Systolic BP/diastolic BP	96 (81–121)	51.50 (37–78)	105.5 (88–124)	52.50 (42–72)	0.002*	0.22
HR peak ex (bpm)	136 (104–155)		126 (106–145)		0.006*	
SV peak ex (mL)	101.2 (64.6–152.2)		114.9 (74.1–142.7)		0.11	
CO peak ex (L/min)	13.5 (9.0–20.3)		14.6 (8.9–18.0)		0.59	
MAP post ex (mmHg)	80.7 (64.3–110.3)		86.3 (72.0–113.3)		0.037*	
Systolic BP/diastolic BP (mmHg)	119 (91–153)	60 (42–96)	126 (105–170)	69 (50–96)	0.06	0.054
ΔHR (%)	40.6 (2.9–29.2)		38.2 (19.0–50.0)		0.09	
ΔSV (%)	24.7 (−6.8–47.7)		28.6 (3.0–47.0)		0.44	
ΔCO (%)	55.6 (26.5–72.9)		55.6 (41.0–66.0)		0.58	

*Note*: Data are presented as median (min–max).

Abbreviations: BP, blood pressure; CHD, pregnant women with congenital heart disease; CO, cardiac output; HR, heart rate; LR, low‐risk pregnancy; MAP, mean arterial pressure; SV, stroke volume.

Three participants were unable to achieve the target HR with exercise. These participants were all in the CHD group. They had the following cardiac procedures: Fontan total cavopulmonary connection, repaired tetralogy of Fallot, and repaired transposition of the great arteries (arterial switch). The last two were on beta‐blockers throughout their pregnancy (Tables 4 and 5).

**TABLE 4:** Characteristics of the participants who were unable to achieve the target heart rate (HR) with exercise.

CHD lesion	Beta‐blockers	Baseline HR (bpm)	Target HR (bpm)	HR at peak exercise (bpm)
Fontan TCPC	No	75	132	102
rToF	Yes (bisoprolol 2.5 mg OD)	78	132	107
rTGA (arterial switch)	Yes (labetalol 100 mg OD)	86	136	123

Abbreviations: bpm, beats/min; OD, once daily; rToF, repaired tetralogy of Fallot; rTGA, repaired transposition of great arteries; TCPC, total cavopulmonary connection.

**TABLE 5:** Doppler indices in the two studied populations.

	LR	CHD	*P*‐value
GA	31 + 6 (30 + 0–34 + 0) (*N* = 62)	32 + 1 (31 + 6–34 + 0) (*N* = 16)	0.040*
UtAD PI	0.698 (0.3–1.7) (*N* = 62)	0.880 (0.6–0.9) (*N* = 3)	0.23
UAD PI	0.965 (0.7–1.4) (*N* = 62)	0.885 (0.7–1.1) (*N* = 16)	0.11
MCA PI	1.970 (1.3–2.9) (*N* = 62)	1.810 (1.5–2.4) (*N* = 16)	0.64
CPR	1.961 (1.3–3.2) (*N* = 62)	2.057 (1.29–2.35) (*N* = 16)	0.91

*Note*: Data are presented as median (min–max).

Abbreviations: CHD, congenital heart disease group; CPR, cerebroplacental ratio; GA, gestational age; LR, low‐risk group; MCA, middle cerebral artery; PI, pulsatility index; UAD, umbilical artery Doppler; UtAD, uterine artery Doppler.

**FIGURE 1:**
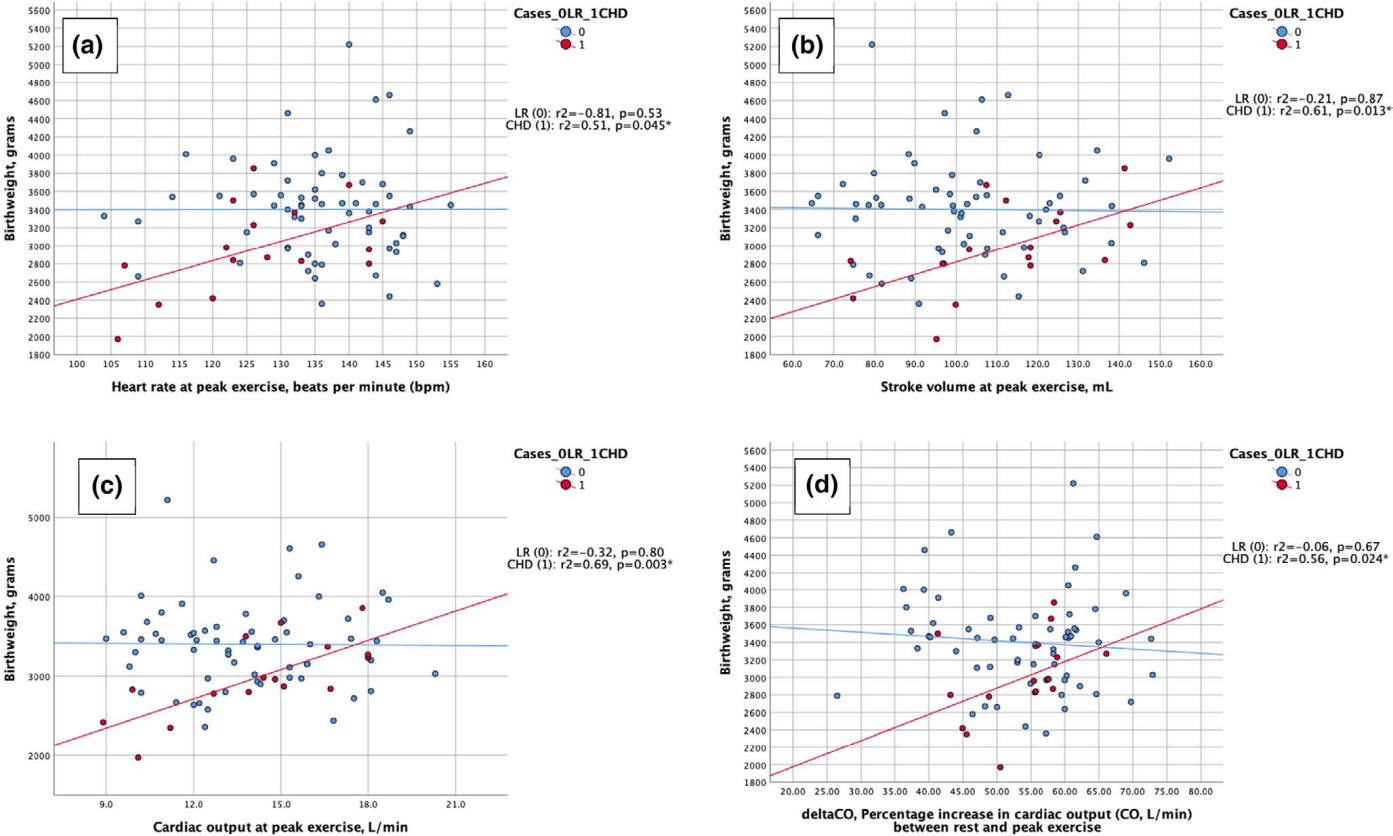
(a) Correlation between heart rate (HR) at peak exercise and birth weight (BW). (b) Correlation between stroke volume (SV) at peak exercise and BW. (c) Correlation between cardiac output (CO) at peak exercise and BW. (d) Correlation between ΔCO and BW. bpm, beats/min.

In the CHD group, there was a significant association between HR at peak exercise (*r*_s_ = 0.51, *P* = 0.045), SV at peak exercise (*r*_s_ = 0.61, *P* = 0.013), CO at peak exercise (*r*_s_ = 0.69, *P* = 0.003), ΔCO (*r*_s_ = 0.56, *P* = 0.024) and BW (Figure 1a–d). When *P*‐values were adjusted for multiple corrections, there was still a statistically significant association between CO at peak exercise and BW (*P* = 0.036).

In the LR group, there was a significant association between peak CO with exercise and EFW (*r*_s_ = 0.29, *P* = 0.043), AC (*r*_s_ = 0.25, *P* = 0.046), and AC/EFW (*r*_s_ = −0.27, *P* = 0.006) (Figure 1e–g). The association remained significant for the association between peak CO with exercise and the AC/EFW ratio after adjusting for multiple corrections (*P* = 0.024). There was also a statistically significant association between HR at rest and BW (*r*_s_ = 0.11, *P* = 0.008) (Figure 1f). This remained significant after adjusting for multiple comparisons (*P* = 0.032).

**FIGURE 2:**
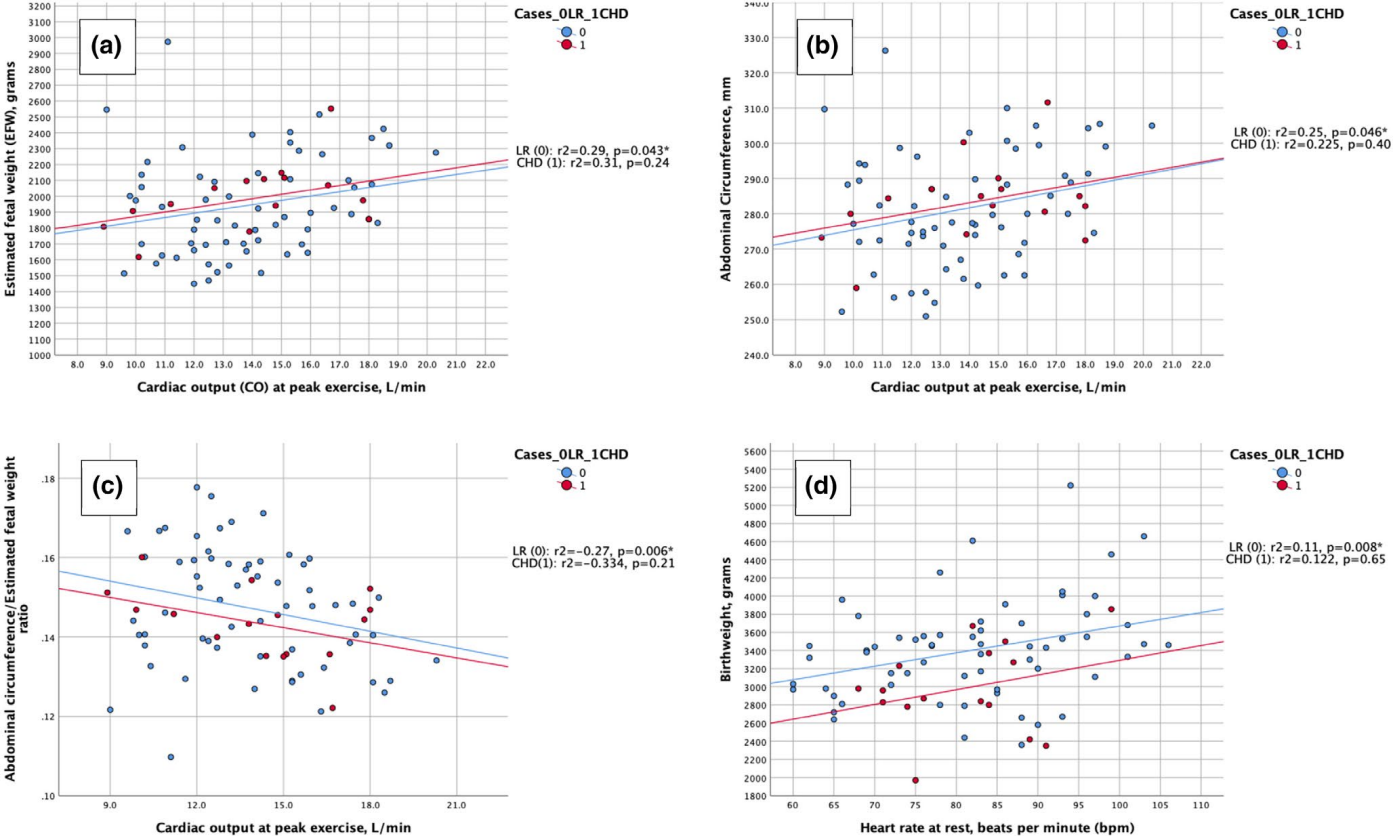
(a) Correlation between cardiac output (CO) at peak exercise and estimated fetal weight (EFW) in the third trimester (30–34 weeks of gestation). (b) Correlation between CO at peak exercise and abdominal circumference (AC) in the third trimester (30–34 weeks of gestation). (c) Correlation between CO at peak exercise and AC/EFW ratio in the third trimester (30–34 weeks of gestation). (d) Correlation between CO at peak exercise and AC/EFW ratio in the third trimester (30–34 weeks of gestation). bpm, beats/min.

**FIGURE 3:**
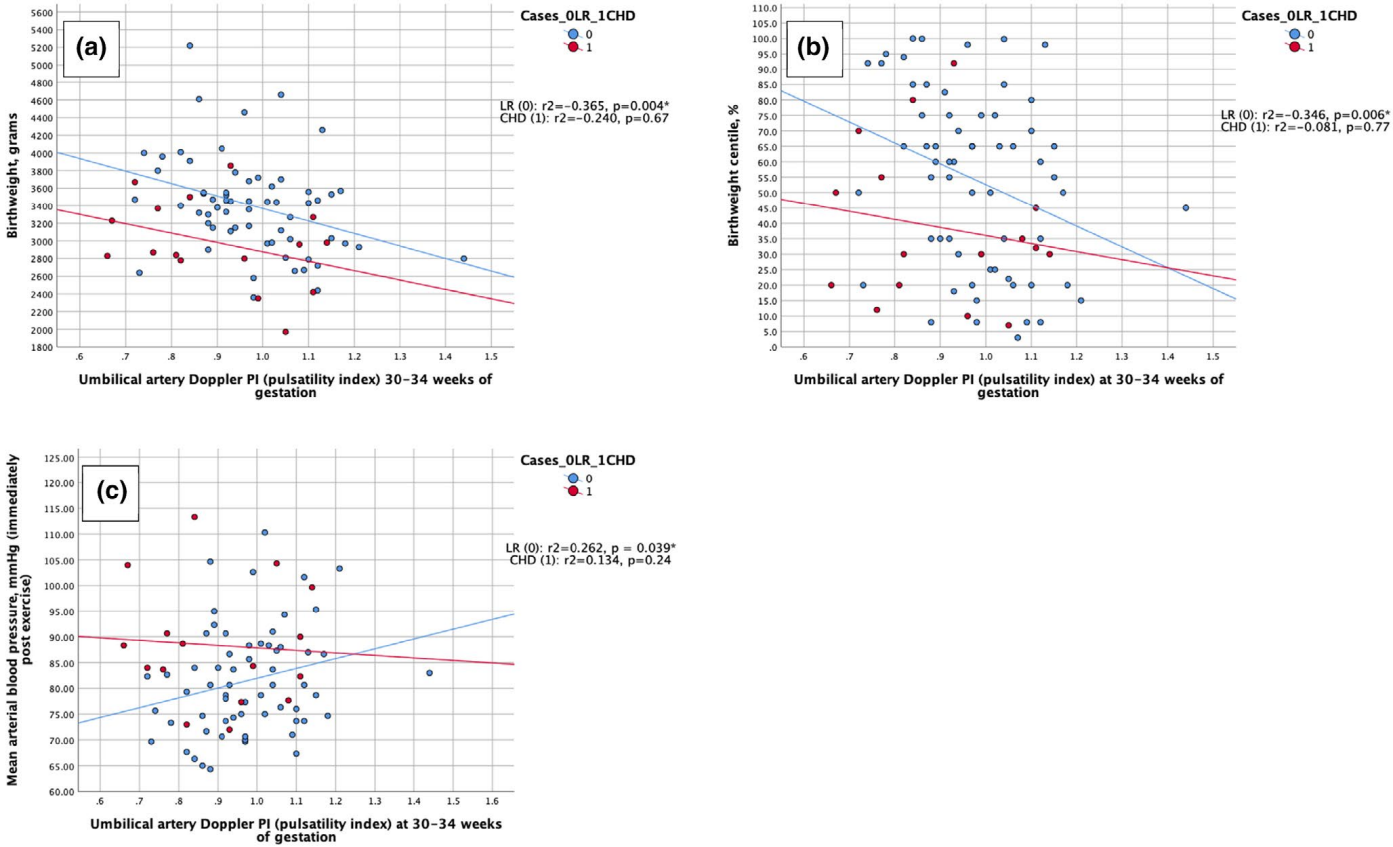
(a) Correlation between umbilical artery Doppler pulsatility index (UAD PI) in the third trimester (30–34 weeks of gestation) and birth weight (BW). (b) Correlation between UAD PI in the third trimester (30–34 weeks of gestation) and BW centile. (c) Correlation between UAD PI in the third trimester (30–34 weeks of gestation) and mean arterial pressure (MAP).

Flow velocity waveform Doppler indices of the two study groups are presented in Figure 2. Median gestational age was higher at measurement of the indices in the CHD group by 2 days (*P* = 0.040). However, using a linear mixed model, all Doppler indices (UtAD pulsatility index [PI], UAD PI, and MCA PI) were corrected for gestational age and no statistically significant difference was found between the two groups (Figure 3).

In the CHD group there were no significant associations between Doppler indices and EFW, AC, BW, or BW centile. In the LR group there were no significant associations between UtAD PI and EFW, AC, BW, or BW centile. There was a statistically significant association between UAD PI and BW (*r*_s_ = −0.365, *P* = 0.004), BW centile (*r*_s_ = −0.346, *P* = 0.006), MCA PI (*r*_s_ = 0.323, *P* = 0.011), and cerebro‐placental ratio (CPR) (*r*_s_ = −0.365, *P* = 0.004) There was also a statistically significant association between CPR and BW (*r*_s_ = 0.383, *P* = 0.002) and BW centiles (*r*_s_ = 0.377, *P* = 0.003). All these associations remained statistically significant after adjusting for multiple comparisons.

In the CHD group, there was no statistically significant association between Doppler indices and cardiac function. In the LR group, there were no statistically significant associations between UtAD PI and HR, SV, CO, or MAP at rest or with exercise. There was a statistically significant association between UAD PI and MAP immediately after exercise (*r*_s_ = 0.262, *P* = 0.039), which, after correcting for multiple testing, was no longer significant.

## 4 DISCUSSION

### 4.1 Summary of main findings

CO at peak exercise was positively associated with BW in the CHD group and negatively associated with the AC/EFW ratio in the LR group, suggesting that adequate placentation translates into a greater ability to increase CO with exercise, facilitating the growth of the fetus.

There were no differences between hemodynamic parameters measured by ICG at rest among the studied populations, and, with peak exercise, only HR was lower in the CHD group. This is a known phenomenon of chronotropic incompetence in this population. MAP was higher in the CHD group at rest and with peak exercise, suggesting an incomplete adaptation to pregnancy.

There were no differences in Doppler indices (UAD and MCA Doppler) between the two studied populations, confirming a somewhat appropriate cardiovascular adaptation in our cohort of surgically corrected CHD patients.

### 4.2 Maternal characteristics and obstetric and fetal outcomes

Participants in the CHD group were years younger and heavier than the LR group. We were unable to perform a subgroup analysis stratifying by age, weight, and BMI, as this would result in small numbers in each of the categories. Women in the CHD group delivered earlier than the LR women (*P* = 0.001), which is consistent with the literature.^2^, ^16^ Women in the CHD group delivered smaller babies. Both poor cardiac function and the use of beta‐blockers have been associated with lower BW.^4^, ^16^ Three of our CHD participants were on beta‐blockers, but no statistical test was performed due to the small sample size.

### 4.3 Maternal hemodynamics

HR at peak exercise was significantly lower in the CHD cohort. Patients with CHD are known to have chronotropic incompetence, an independent predictor of mortality.^17^ A reduced HR response to exercise in pregnant women with CHD might affect their ability to increase CO as pregnancy progresses and potentially increase the risk of cardiac events.^17^

Mean arterial pressures at rest and immediately after exercise were significantly higher in the CHD participants, mainly due to higher systolic BP. There were no differences in SV or CO at rest or at peak exercise, suggesting a somewhat appropriate cardiovascular adaptation to pregnancy in women with CHD. In women with CHD, cardiovascular adaptation to pregnancy is attenuated.^1^ Our CHD participants were in functional class 1–2 of the New York Heart Association (NYHA), which may explain the minimal differences in the cardiovascular parameters between populations. Cardiac function was measured using ICG, which may have affected the results, although this device has been validated for clinical use in the non‐pregnant population.^18^, ^19^, ^20^, ^21^, ^22^

### 4.4 Association between cardiac function and fetal size

In both groups, those with higher CO at peak exercise had higher EFW and larger AC, demonstrating a clear relationship between CO and fetal weight. Although statistical significance was not reached for the CHD group, likely due to the small number of participants, the correlation curves were similar in both groups.

We saw a significant association between maternal HR, SV, and CO at peak exercise in the CHD group and BW, with those with a higher ability to increase HR, SV, and CO having larger babies. Bamfo et al.^14^ showed that in pregnancies complicated by FGR, CO was lower and TVR was higher than in normal pregnancies. In another study, Bamfo et al.^23^ described similar findings and suggested that a logistic regression equation incorporating maternal echocardiographic parameters could be used to diagnose FGR. Vasapollo et al.^13^ also showed that women with FGR had lower CO and higher MAP and TVR compared with women with babies that were SGA but not growth‐restricted. The authors suggested that maternal cardiovascular dysfunction precedes and is a contributor to the development of FGR and that TVR might be considered an indirect marker of cardiovascular function in pregnancy.^13^

In our cohort, women in the CHD group had significantly smaller babies and higher MAP, which is consistent with the studies described here, but we were not able to demonstrate lower CO in the CHD population, possibly because of small numbers or due to limitations in ICG.

### 4.5 Doppler indices

There were no differences between the two groups in any of the Doppler indices studied. Pieper et al.,^24^ who performed Doppler studies in about 150 pregnant women with CHD (compared with 70 LR pregnancies) showed that both UtAD and UAD PIs were significantly higher in the CHD group at 20 and 32 weeks of gestation, and that a high UAD PI at 32 weeks was associated with “obstetric events” in the CHD group. Goya et al.^25^ also showed increased UtAD PI and increased UAD PI in 170 pregnant women with heart disease (of whom 70% had CHD; compared with 75 LR pregnancies) and a raised UAD PI at 32 weeks of gestation was associated with “obstetric events.” Obstetric events in these studies were a combination of outcomes such as SGA/FGR and pre‐eclampsia. Both these studies included participants with a broad mixture of CHD lesions in different functional classes.

Although we could not demonstrate an association between UtAD and fetal size, we found a significant correlation between UAD PI and BW and BW centile in the LR group, with those with higher resistance delivering smaller babies.

Kampman et al.^26^ reported in their systematic review that women with heart disease have a higher incidence of abnormalities in utero‐placental blood flow indices and poor obstetric outcome and, at the same time, women with abnormal utero‐placental flow are also more likely to have cardiac dysfunction.^27^ The placentation process produces a low‐resistance system in the utero‐placental interface, and blood flow depends mainly on maternal cardiovascular function due to the absence of autoregulation in the utero‐placental interface.^27^

Our data do not support the studies described here. However, we only had data on UtAD for three participants in the CHD group, which prevented a meaningful analysis, and the number of CHD participants with data on UAD was also small compared with the LR group (*n* = 16 vs. *n* = 62), which could explain these findings. The CHD lesions were multiple (11 different lesions in 16 participants), which could cause scatter of the data as different cardiac lesions will have different hemodynamic implications.

### 4.6 Doppler indices and association with other variables

Our median BW centile, for the LR population, was 60.0 and only four out the 62 participants had BW centiles below the 10th centile. None had notching of UtAD and only one had a mean UtAD PI >1.45. None developed pre‐eclampsia and only one participant developed gestational hypertension at term. This makes our population essentially LR, which might explain the lack of association between UtAD and CO, SV, EFW, BW, and BW centiles.

In both groups, those with higher PI index in the UAD delivered smaller babies with lower BW and lower BW centiles, although statistical significance was only reached for the LR group.

## AUTHOR CONTRIBUTIONS

F.D.S: conceptualization, methodology, formal analysis, investigation, writing—original draft. E.B: writing—review and editing. P.S: formal analysis, writing—review and editing, supervision. M.J: review and editing, supervision.

## FUNDING INFORMATION

BORNE Charity (UK registration number: 1167063) who provided funding for all consumables used in this study.

## CONFLICT OF INTEREST STATEMENT

The authors have no conflicts of interest.

## Data Availability

The data that support the findings of this study are available from the corresponding author, FDS, upon reasonable request.

## References

[1] Kampman MA , Valente MA , van Melle JP , et al. Cardiac adaption during pregnancy in women with congenital heart disease and healthy women. Heart. 2016;102(16):1302‐1308. doi:10.1136/heartjnl-2015-308946 27048772

[2] Schlichting LE , Insaf TZ , Zaidi AN , Lui GK , Van Zutphen AR . Maternal comorbidities and complications of delivery in pregnant women with congenital heart disease. J Am Coll Cardiol. 2019;73(17):2181‐2191. doi:10.1016/j.jacc.2019.01.069 31047006

[3] Roos‐Hesselink J , Baris L , Johnson M , et al. Pregnancy outcomes in women with cardiovascular disease: evolving trends over 10 years in the ESC registry of pregnancy and cardiac disease (ROPAC). Eur Heart J. 2019;40:3848‐3855. doi:10.1093/eurheartj/ehz136 30907409

[4] Cauldwell M , Steer P , Sterrenburg M , et al. Birth weight in pregnancies complicated by maternal heart disease. Heart. 2019;105:391‐398. doi:10.1136/heartjnl-2018-313551 30242140

[5] Cornette J , Ruys TP , Rossi A , et al. Hemodynamic adaptation to pregnancy in women with structural heart disease. Int J Cardiol. 2013;168(2):825‐831. doi:10.1016/j.ijcard.2012.10.005 23151412

[6] Bamfo JE , Kametas NA , Chambers JB , Nicolaides KH . Maternal cardiac function in normotensive and pre‐eclamptic intrauterine growth restriction. Ultrasound Obstet Gynecol. 2008;32(5):682‐686. doi:10.1002/uog.5311 18702086

[7] Ling HZ , Guy GP , Bisquera A , Poon LC , Nicolaides KH , Kametas NA . Maternal hemodynamics in screen‐positive and screen‐negative women of the ASPRE trial. Ultrasound Obstet Gynecol. 2019;54(1):51‐57. doi:10.1002/uog.20125 30246326

[8] Balci A , Sollie KM , Mulder BJ , et al. Associations between cardiovascular parameters and uteroplacental doppler (blood) flow patterns during pregnancy in women with congenital heart disease: rationale and design of the Zwangerschap bij Aangeboren Hartafwijking (ZAHARA) II study. Am Heart J. 2011;161(2):269‐275. doi:10.1016/j.ahj.2010.10.024 21315208

[9] Salomon LJ , Alfirevic Z , Da Silva Costa F , et al. ISUOG practice guidelines: ultrasound assessment of fetal biometry and growth. Ultrasound Obstet Gynecol. 2019;53(6):715‐723. doi:10.1002/uog.20272 31169958

[10] Bhide A , Acharya G , Bilardo CM , et al. ISUOG practice guidelines: use of doppler ultrasonography in obstetrics. Ultrasound Obstet Gynecol. 2013;41(2):233‐239. doi:10.1002/uog.12371 23371348

[11] de Greeff A , Beg Z , Gangji Z , Dorney E , Shennan AH . Accuracy of inflationary versus deflationary oscillometry in pregnancy and preeclampsia: OMRON‐MIT versus OMRON‐M7. Blood Press Monit. 2009;14(1):37‐40. doi:10.1097/MBP.0b013e32831e305d 19252437

[12] Chung Y , Brochut MC , de Greeff A , Shennan AH . Clinical accuracy of inflationary oscillometry in pregnancy and pre‐eclampsia: Omron‐MIT elite. Pregnancy Hypertens. 2012;2(4):411‐415. doi:10.1016/j.preghy.2012.04.001 26105612

[13] Vasapollo B , Valensise H , Novelli GP , Altomare F , Galante A , Arduini D . Abnormal maternal cardiac function precedes the clinical manifestation of fetal growth restriction. Ultrasound Obstet Gynecol. 2004;24(1):23‐29. doi:10.1002/uog.1095 15229912

[14] Bamfo JE , Kametas NA , Turan O , Khaw A , Nicolaides KH . Maternal cardiac function in fetal growth restriction. BJOG. 2006;113(7):784‐791. doi:10.1111/j.1471-0528.2006.00945.x 16827761

[15] Wallace JM , Bhattacharya S , Horgan GW . Gestational age, gender and parity specific centile charts for placental weight for singleton deliveries in Aberdeen, UK. Placenta. 2013;34(3):269‐274. doi:10.1016/j.placenta.2012.12.007 23332414

[16] Gelson E , Curry R , Gatzoulis MA , et al. Effect of maternal heart disease on fetal growth. Obstet Gynecol. 2011;117(4):886‐891. doi:10.1097/AOG.0b013e31820cab69 21422861

[17] Lui GK , Silversides CK , Khairy P , et al. Heart rate response during exercise and pregnancy outcome in women with congenital heart disease. Circulation. 2011;123(3):242‐248. doi:10.1161/CIRCULATIONAHA.110.953380 21220738

[18] Bour J , Kellett J . Impedance cardiography: a rapid and cost‐effective screening tool for cardiac disease. Eur J Intern Med. 2008;19(6):399‐405. doi:10.1016/j.ejim.2007.07.007 18848172

[19] Charloux A , Lonsdorfer‐Wolf E , Richard R , et al. A new impedance cardiograph device for the non‐invasive evaluation of cardiac output at rest and during exercise: comparison with the "direct" Fick method. Eur J Appl Physiol. 2000;82(4):313‐320. doi:10.1007/s004210000226 10958374

[20] Richard R , Lonsdorfer‐Wolf E , Charloux A , et al. Non‐invasive cardiac output evaluation during a maximal progressive exercise test, using a new impedance cardiograph device. Eur J Appl Physiol. 2001;85(3–4):202‐207. doi:10.1007/s004210100458 11560071

[21] Broeder C , Hickock J , Burditt A . Does Advanced Cardiac Impedance Technology Accurately Measure Cardiac Output during Submaximal Steady State Exercise? (Scientific Abstract). Benedictine University. 2009.

[22] Robach P , Calbet JA , Thomsen JJ , et al. The ergogenic effect of recombinant human erythropoietin on VO2max depends on the severity of arterial hypoxemia. PLoS One. 2008;3(8): e2996. doi:10.1371/journal.pone.0002996 18714372 PMC2500186

[23] Bamfo JE , Kametas NA , Chambers JB , Nicolaides KH . Maternal cardiac function in fetal growth‐restricted and non‐growth‐restricted small‐for‐gestational age pregnancies. Ultrasound Obstet Gynecol. 2007;29(1):51‐57. doi:10.1002/uog.3901 17200990

[24] Pieper PG , Balci A , Aarnoudse JG , et al. Uteroplacental blood flow, cardiac function, and pregnancy outcome in women with congenital heart disease. Circulation. 2013;128(23):2478‐2487. doi:10.1161/CIRCULATIONAHA.113.002810 24192800

[25] Goya M , Casellas M , Merced C , et al. Predictors of obstetric complications in women with heart disease. J Matern Fetal Neonatal Med. 2016;29(14):2306‐2311. doi:10.3109/14767058.2015.1085012 26371393

[26] Kampman MA , Siegmund AS , Bilardo CM , et al. Uteroplacental doppler flow and pregnancy outcome in women with tetralogy of Fallot. Ultrasound Obstet Gynecol. 2017;49(2):231‐239. doi:10.1002/uog.15938 27071979

[27] Kampman MA , Bilardo CM , Mulder BJ , et al. Maternal cardiac function, uteroplacental doppler flow parameters and pregnancy outcome: a systematic review. Ultrasound Obstet Gynecol. 2015;46(1):21‐28. doi:10.1002/uog.14697 25320041

